# Establishing an Elective Rotation Director and Its Effect on Elective Opportunities and Satisfaction

**DOI:** 10.5811/westjem.2019.10.44111

**Published:** 2019-12-09

**Authors:** Adam J. Janicki, Michele L. Dorfsman

**Affiliations:** University of Pittsburgh School of Medicine, Department of Emergency Medicine, Pittsburgh, Pennsylvania

## Abstract

Elective rotations are valuable, allowing trainees to personalize their educational experience, focus on areas of weakness, and offer personal and professional development. Emergency medicine (EM) residency program elective rotations may be limited due to the absence of awareness of opportunities and administrative support. We sought to increase the breadth of elective rotation opportunities, improve residents’ satisfaction with their elective rotations, and enhance the opportunities for clinical training. To increase the breadth of our elective rotation opportunities, we established an elective rotation director—a dedicated faculty member to aid in elective planning and provide administrative support. This faculty member met with all residents during their second year, coordinated new electives with the graduate medical education office, and assisted with administrative tasks. Ten new rotations (two local, five domestic away, three international away) were established during the position’s first two years, increasing available rotations from nine to 19. A survey was sent to graduates of the program two years before and two years after the position was established to inquire about their elective experience. Of 64 graduates, 49 (76.6%) participated in the survey. Graduates exposed to the dedicated faculty member reported increased exposure to novel learning environments (p<0.001), improved wellness (p<0.001), and were more likely than pre-director graduates to choose the same elective rotation (p=0.006). Programs with multiple elective rotations may benefit more from this position, but additional resources may be needed given the associated increase in administrative time.

## BACKGROUND

The majority of emergency medicine (EM) residency programs in the United States (U.S.) offer opportunities for local, domestic and international elective rotations. Elective rotations allow trainees to personalize their educational experience, focus on areas of weakness, and provide opportunities for personal and professional development.^1^,^2^ Increased interest in domestic and international away rotations has placed more pressure on residency programs to offer a wide variety of elective opportunities.^3^ International rotations have been previously shown to be a source of resident satisfaction and a strong recruitment tool for EM residency programs; however, significant barriers remain to expand these opportunities.^4^

Over the last five years, our residents consistently cited an absence of opportunity awareness and administrative support as barriers to the creation of new local electives and pursuit of away-elective opportunities. Local and institutional barriers made navigating the process daunting for residents, even with the help of program coordinators tasked with facilitating elective rotations. This often resulted in our residents often limiting themselves to local opportunities in areas such as ultrasound, research, emergency medical services, and toxicology.

## OBJECTIVES

We sought to increase the breadth of elective rotation opportunities our training program offers, improve resident satisfaction with their elective rotations, and enhance opportunities for clinical training.

## DESIGN

In October 2016 we established an elective rotation director, a dedicated academic faculty member to aid in elective planning and provide administrative support. This faculty mentor, in conjunction with the residency program director (PD), worked with each resident to expand and individualize elective rotation choices to include more local, national, and international rotations.

Our residency program is a three-year program that offers only one elective rotation during the third year. The faculty member met with each resident early in postgraduate year (PGY)-2 to identify three viable rotation options based upon the resident’s interests. The director subsequently helped the resident procure his or her ideal rotation while ensuring that backup choices were still attractive. The elective rotation director worked with the residency PD, the rotation director at the receiving institution for away rotations, and the graduate medical education (GME) office to complete all administrative tasks including completion of program letters of agreement (PLAs), memorandums of understanding, and master affiliation agreements. At the end of the academic year, the faculty member presented a summary of elective opportunities at residency education conference to inform junior residents for future planning.

The rotation director met with the residency program leadership monthly to review established rotations, revise new rotations based on feedback, and to ensure all institutional and national residency guidelines were followed. While administrative support from program coordinators is essential, the process is optimized through guidance that a faculty member provides in craftign goals and objectives and ensuring educational value. The rotation director received a financial stipend and adequate protected time to meet with each resident, research elective opportunities based on the resident’s academic wants and needs, conceptualize rotations and ensure they met institutional guidelines, and complete administrative tasks.

Much was learned from the position’s creation and implementation. The largest, unexpected barriers were administrative. Much time was spent revising PLAs based on departmental and Office of Graduate Medical Eduation feedback. Away rotations, both domestic and international, and planning and administrative tasks were delayed based upon feedback and response times from hosting institutions. We established that it takes approximately six months from elective conceptualization to finalization and confirmation.

## IMPACT/EFFECTIVENESS

To assess the impact of the director, we determined the number of elective rotation opportunities two years prior to (pre-director) and two years after (post-director) the creation and implementation of the director position. In addition, a survey designed by the study authors to obtain preliminary data regarding the position’s effectiveness was distributed directly to both pre-director and post-director graduates (Supplemental File). The survey addressed the following domains: resident satisfaction; electives’ learning environments, wellness, and attitudes towards the elective rotation director position. We used five-point Likert scale items in which the lowest score (1) corresponded to “strongly disagree” and the highest score (5) corresponded to “strongly agree.” The survey was reviewed by two experts in medical education as well as an expert in survey design to ascertain content validity. The survey was piloted on a resident physician not participating in the study to assess response process validity and was subsequently revised based on feedback.

Residency graduates who participated in an elective rotation during their residency training were emailed invitations directly with a survey link administered via SurveyMonkey. Student’s t-test was used to compare responses and effect sizes were calculated (5,6). Given our 1–5 Likert scale, effect sizes greater than 0.5 were deemed large, 0.25–0.5 intermediate, and less than 0.25 small. All study procedures were approved by the University of Pittsburgh Institutional Review Board.

Prior to the elective rotation director, nine rotations were offered – seven local (within our institution), one domestic away (within the U.S., but outside of our institution), and one international away rotation. Two years after establishing this position, elective opportunities increased to 19 rotations – nine local, six domestic away, and four international away. Table 1 provides a description of offered electives.

Our survey was completed by 49 of 64 (76.6%) of eligible graduates, 20 pre-director graduates (62.5%), and 29 post-director graduates (90.6%). Post-director graduates felt that their elective exposed them to a novel learning environment (p<0.001, effect size = 1.15) and contributed to their wellness (p<0.001, effect size = 1.08). If offered the opportunity to choose their elective again, post-director graduates were more likely than pre-director to choose the same elective rotation (p=0.006, effect size = 0.77). Pre-director graduates reported that they would have welcomed administrative support in planning their elective rotation (mean 3.86; 95% confidence interval [CI], 3.4–4.5), and post-director graduates felt that administrative support helped them plan their elective rotation (mean 4.5; 95% CI, 4.3–4.7).

Programs with multiple elective rotations may benefit even more from this position given the associated increase in planning and burden of administrative tasks. Additional resources may be needed given the associated increase in required protected time.

This position was implemented at a single institution with results from a small cohort of residency graduates, but preliminary data supports its creation given the increased breadth of available elective opportunities and potential impact on resident education and well-being.

## Supplementary Information



## Figures and Tables

**Table 1:** Elective rotations including location and description of away electives.

	Pre-director^a^	Post-director^b^
Local	Clinical decision unit/observation	Orthopedics
	Emergency medical services	Sports medicine
	Medical education	
	Post-cardiac arrest service	
	Research	
	Toxicology	
	Ultrasound	
Domestic Away	Honolulu, Hawaii	Anchorage, Alaska
	Emergency medicine	Emergency medicine
		Native American medical care
		Block Island, Rhode Island
		Island medicine
		Rural medicine
		Denver, Colorado
		Wilderness medicine
		Medical education
		Telluride, Colorado
		Emergency medicine
		Wilderness medicine
		Tuba City, Arizona
		Rural medicine
		Native American medical care
International Away	Auckland, New Zealand	Cape Town, South Africa
	Emergency medicine	Emergency medicine
		Bali, Indonesia
		Medical education
		Tropical medicine
		American Samoa
		Emergency medicine
		Tropical medicine

a Electives offered prior to establishing director position.

b Electives added after establishing director position based on resident interest.

## References

[1] Hayden SR, Valderrama CM, Xu M (2016). Development of an international elective in an emergency medicine residency. J Emerg Med.

[2] Gottlieb M, Arno K, Kuhns M (2018). Distribution of clinical rotations among emergency medicine residency programs in the United States. AEM Educ Train.

[3] Hansoti B, Douglass K, Tupesis J (2013). Guidelines for Safety of Trainees Rotating Abroad: Consensus Recommendations from the Global Emergency Medicine Academy of the Society for Academic Emergency Medicine, Council of Emergency Medicine Residency Directors, and the Emergency Medicine Residents’ Association. Acad Emerg Med.

[4] Drain P, Primack A, Hunt D (2007). Global health in medical education: a call for more training and opportunities. Acad Med.

[5] Lenhard W, Lenhard A (2016). Calculation of Effect Sizes.

[6] Cohen J (1988). Statistical power analysis for the behavioral sciences.

