# Supplementary material for: Establishing an Elective Rotation Director and Its Effect on Elective Opportunities and Satisfaction
Source: West J Emerg Med. 2019 Dec 9;21(1):8–11. doi: 10.5811/westjem.2019.10.44111 (PMC6948683; doi:10.5811/westjem.2019.10.44111)
Supplement: Supplementary file 1 [file wjem-21-8-s001.docx]

Year you graduated from UPMC Emergency Medicine Residency

2016

2017

2018

2019

Where did you do your elective rotation:

Local site

Domestic away

International away rotation (outside of the continental US, Hawaii, or Alaska)

How would you classify your elective rotation? Select all that apply.

Administration

Clinical

Education and/or teaching

Research

Ultrasound (EM)

Other: ______________________________

If you did a clinical elective, on what service did you rotate? Select all that apply.

Critical Care

Emergency Medicine

EMS - ground

EMS - air

Ophthalmology

Orthopedics

Pediatrics

Radiology

Rural/critical access

Toxicology

Ultrasound (EM)

Wilderness Medicine

Other: _____________________

N/A

If you did a research elective, did your research elective lead to: (select all that apply)

A manuscript publication

A research grant

Neither

If you did an education and/or teaching elective, did your education and/or teaching elective lead to the creation of any of the following: (select all that apply)

A learning module

A new curriculum or change in curriculum

A publication

None of the above

My elective rotation gave me exposure to a learning environment that I was otherwise not exposed to during residency.

Strongly disagree

Disagree

Neither agree nor disagree

Agree

Strongly agree

My elective rotation improved my clinical knowledge.

Strongly disagree

Disagree

Neither agree nor disagree

Agree

Strongly agree

My elective rotation improved my clinical skills.

Strongly disagree

Disagree

Neither agree nor disagree

Agree

Strongly agree

My elective helped me complete my scholarly work (learning module, curriculum, and/or publication)

Strongly disagree

Disagree

Neither agree nor disagree

Agree

Strongly agree

My elective rotation contributed to my wellness.

Strongly disagree

Disagree

Neither agree nor disagree

Agree

Strongly agree

My elective rotation decreased my burnout.

Strongly disagree

Disagree

Neither agree nor disagree

Agree

Strongly agree

If given the option again I would do the SAME elective rotation.

Strongly disagree

Disagree

Neither agree nor disagree

Agree

Strongly agree

If given the option again I would do a DIFFERENT elective rotation.

Strongly disagree

Disagree

Neither agree nor disagree

Agree

Strongly agree

Administrative support would have helped me while planning my elective rotation.

Strongly disagree

Disagree

Neither agree nor disagree

Agree

Strongly agree

N/A (I had administrative support)

If I had administrative support in planning my elective, I would have done a DIFFERENT elective rotation.

Strongly disagree

Disagree

Neither agree nor disagree

Agree

Strongly agree

N/A (I had administrative support)

If I had administrative help I would have pursued an away elective rotation.

Strongly disagree

Disagree

Neither agree nor disagree

Agree

Strongly agree

N/A (I had administrative support)

Administrative support/guidance helped me while planning my elective rotation.

Strongly disagree

Disagree

Neither agree nor disagree

Agree

Strongly agree

N/A (I did not have administrative support)

How would you describe your current job? (select all that apply)

Community EM

Academic EM

Fellowship

Other (please specify):_______________

Please provide any additional comments regarding your elective.

Age:

Gender:

Man

Woman

Non-binary gender

Which of the following best describes your current relationship status?

Single/never married

Married/civil partnership

Separated

Divorced

Widowed

Other, specify:

How do you self-identify? (select all that apply)

American Indian or Alaska Native

Asian

Black or African American

Hispanic, Latino, or of Spanish origin

Native Hawaiian or Other Pacific Islander

White

Other______________
